# Combating infectious disease outbreaks in Somalia’s fragile health system: the impact of climate change-narrative review

**DOI:** 10.1186/s41182-025-00816-3

**Published:** 2025-10-23

**Authors:** Saadaq Adan Hussein, Marian Muse Osman, Mohamed Mohamoud Hassan, Mohamed Abdullahi Awale, Yahye Sheikh Abdulle Hassan, Abdullahi Mohamed Mohamud, Abdirahman Aden Hussein, Tahlil Abdi Afrah, Abdirahman Moallim Ibrahim, Abdinafic Mohamud Hussein, Khadar Hussein Mohamud, Abdinur Hussein Mohamed, Rage Adem, Mohamed MAli Fuje, Abdullahi Ali Hayle, Walid Abdulkadir Osman, AbdulJalil Abdullahi Ali, Ayan Nur Ali, Chukwuma David Umeokonkwo

**Affiliations:** 1 Department of School of Postgraduate Studies, Benadir University, Mogadishu, Somalia; 2 Department of Social and Human Capital Development Pillar, Office of the Prime Minister, Federal Republic, Mogadishu, Somalia; 3 Department of Research and Policy Development, SOR Institute: Somalia Social Research, Mogadishu, Somalia; 4 Department Benadir Institute for Research and Development, Benadir University, Mogadishu, Somalia; 5 Department Research, Somali National Institute of Health, Mogadishu, Somalia; 6 Department Rector, Benadir University, Mogadishu, Somalia; 7 Department Faculty of Medicine and Surgery, Benadir University, Mogadishu, Somalia; 8 Department Faculty of Medicine and Surgery, Jamhuriya University of Science and Technology, Mogadishu, Somalia; 9 Department Faculty of Medicine and Surgery, Jazeera University, Mogadishu, Somalia; 10 Department Executive of Somali Development Research Institute (SODRI), Mogadishu, Somalia; 11 Department of Faculty of Health Sciences, Benadir University, Mogadishu, Somalia; 12 Department of Faculty of Health Sciences, Mogadishu University, Mogadishu, Somalia; 13 Department of Dialysis, Mogadishu Somali Türkiye Training and Research Hospital, Mogadishu, Somalia; 14 Department African Field Epidemiology Network, Kampala, Uganda

**Keywords:** Somalia, Climate change, Cholera, Measles, Polio, Fragile health system, Narrative review

## Abstract

**Introduction:**

Somalia, the 44th largest country in the world by land area, struggles with a heavy burden of infectious diseases. Since 1991, populations have lacked essential health services, exacerbated by recurring infectious-disease outbreaks. Recurrent outbreaks of measles, cholera, and polio have devastated public health, generating significant morbidity and mortality. Despite improvements through new graduates, these issues remain unresolved. This study examines the impact of climate change on infectious-disease outbreaks in Somalia focusing on cholera, measles, and polio—to fill a gap in the literature by linking climate variability with outbreak dynamics and identifying weaknesses in Somalia’s health system. The findings will inform targeted public-health strategies.

**Method:**

Following PRISMA guidelines, we undertook a narrative review of English-language literature (1990 – March 2025). Searches in PubMed, Scopus, Web of Science and Google Scholar combined terms for infectious-disease outbreaks, climate change and Somalia/Horn of Africa. Of 202 records identified, 74 met inclusion criteria. Two reviewers independently screened, extracted data and applied six-step inductive coding in NVivo 12, synthesizing findings into thematic domains.

**Results:**

Four interlinked themes emerged. (1) Fragile health system: < 0.4 doctors, nurses and midwives per 10 000 population, poorly equipped facilities and patchy surveillance. (2) Control measures: routine immunization completeness ≈20%; limited oral-cholera-vaccine and WASH coverage sustain transmission. (3) Political instability and conflict: insecurity, decentralized coordination and ≥ 2.6 million IDPs hamper rapid response. (4) Impact of climate change: drought-induced water scarcity and flood-related latrine breaches create year-round face-oral exposure, while climate shocks divert resources and swell susceptibility pools.

**Conclusion:**

Outbreak control in Somalia now hinges on integrating climate adaptation with health-system strengthening. Climate-proofed WASH infrastructure, mobile vaccination and surveillance linked to hydro-meteorological alerts, a National Outbreak Operations Centre, and ring-fenced financing are urgent priorities. Without such measures each extreme-weather event will erase hard-won gains; with them, Somalia can break the climate–outbreak feedback loop.

## Background

Somalia, the 44th largest country in the world by land area [1, 2], has been plagued by conflict, displacement, climate change, and struggles with a heavy burden of infectious diseases [3]. Since 1991, conflict, displacement, and droughts have left Somalia's vulnerable populations without essential health services, exacerbated by recurring infectious disease outbreaks, including one in early 2024[4, 5], and has been grappling with one of the world's longest-running humanitarian crisis [6–8]. The civil war that erupted in 1990 destroyed Somalia's health infrastructure [9], with a severe impact on the health and livelihood of millions of people [10, 11].

**Fig. 1:**
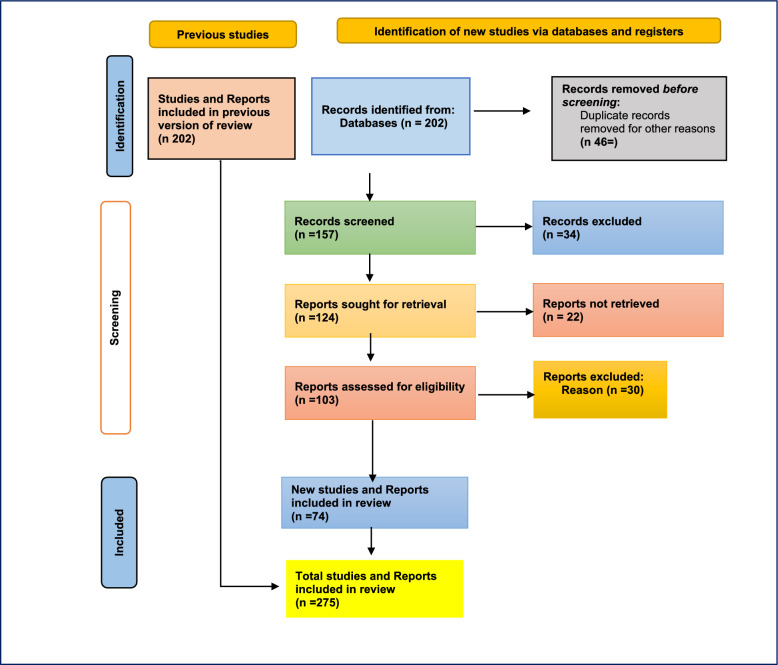
Recurring Infectious-Disease Outbreaks in Somalia, 1990–2025 (Polio, Measles, Cholera)

Recurrent outbreaks of measles, cholera, and polio have devastated public health, with Somalia consistently [12, 13] ranking among the lowest in global health indicators [14]. Historically, outbreaks have generated significant morbidity and mortality, disrupted essential services, and curtailed socio-economic development, underscoring persistent fragilities in disease control [15]. Cholera remains a major public health issue, worsened by flooding, poor sanitation, and displacement, emphasizing the need for improved water and sanitation infrastructure [16, 17]. Recent global outbreaks, such as Lebanon’s 2022 cholera resurgence [18] and Malawi’s Cyclone Freddy surge [19], highlight the impact of climate on disease spread and the need for resilient systems in Somalia. Kenya’s success in cholera containment through surveillance and rapid WASH upgrades provides lessons for Somalia [20]. Figure 1 provides an overview of recurring infectious disease outbreaks in Somalia from 1990 to 2025, including polio, measles, and cholera.

**Fig. 2:**
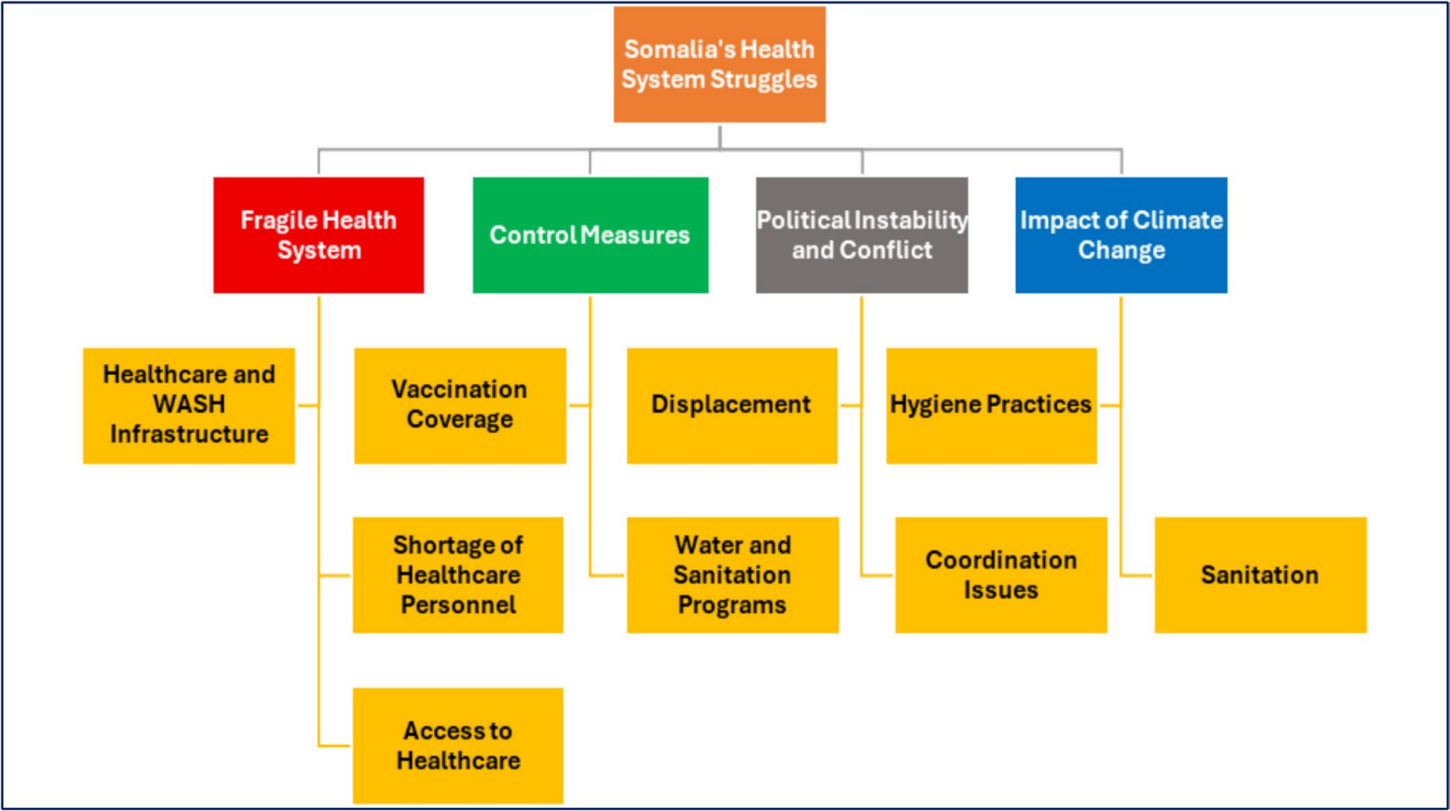
Annual Measles Cases and Incidence Rate in Somalia, 2000 – 2023, by Administrative, Official, and WHO [68]

**Fig. 3:**
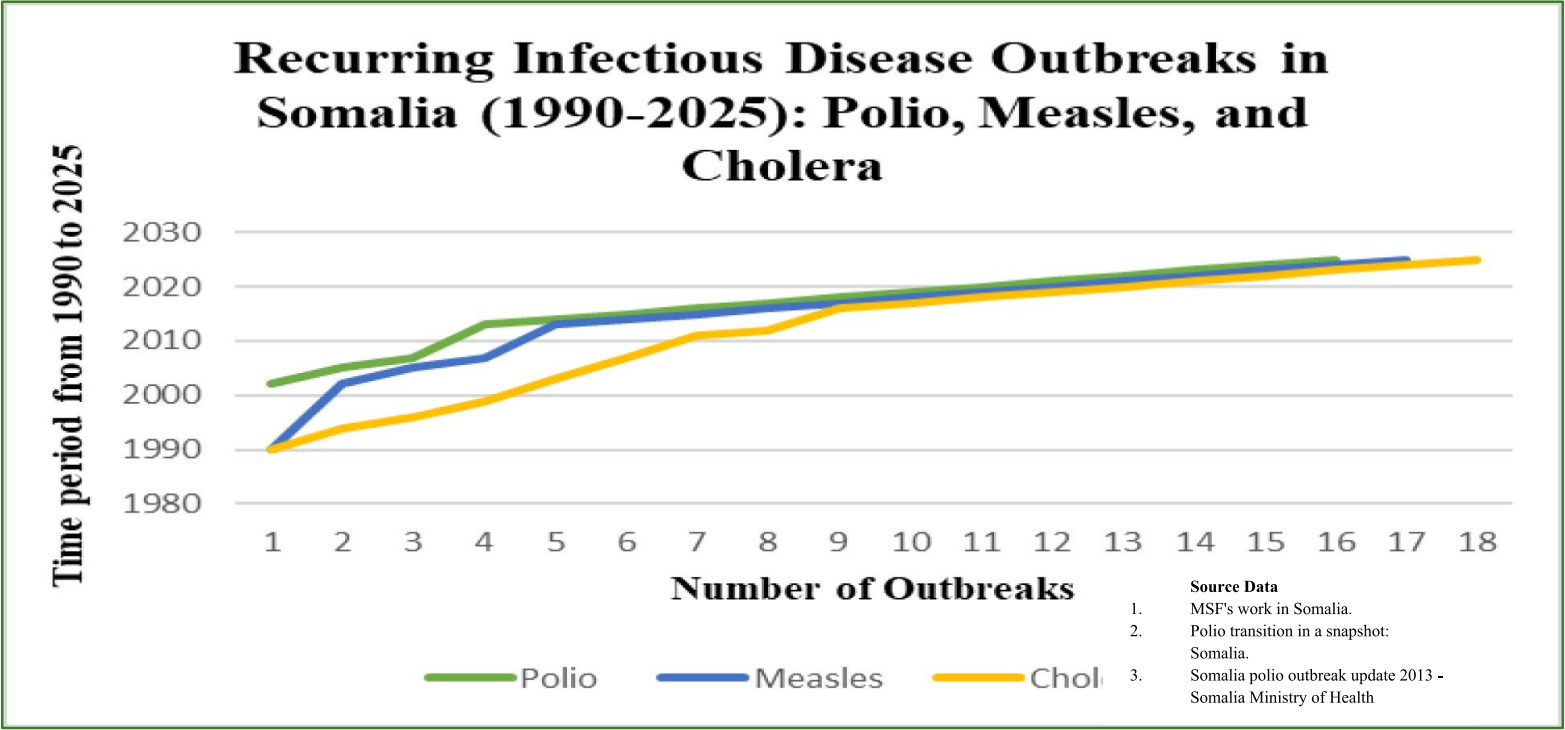
Trends in Polio Third-Dose Vaccination Coverage in Somalia, 2000 – 2023, by Administrative, Official, and WHO/UNICEF Estimates [68]

Measles is endemic, with frequent outbreaks exacerbated by limited vaccine access and a fragile healthcare system [21]. Figure 2 presents annual measles cases and incidence rates in Somalia from 2000 to 2023, based on administrative, official, and WHO data. Polio transmission remains a significant concern despite efforts to control it [22]. The COVID-19 pandemic revealed structural deficits in disease surveillance and vaccination systems, worsening polio and other health challenges [23]. Figure 3 displays trends in polio third-dose vaccination coverage in Somalia, from 2000 to 2023, using official and WHO/UNICEF estimates. The COVID-19 pandemic highlighted the critical need for more strategic deployment of oral cholera vaccines (OCV) in response to increasing disease outbreaks [24].

**Fig. 4:**
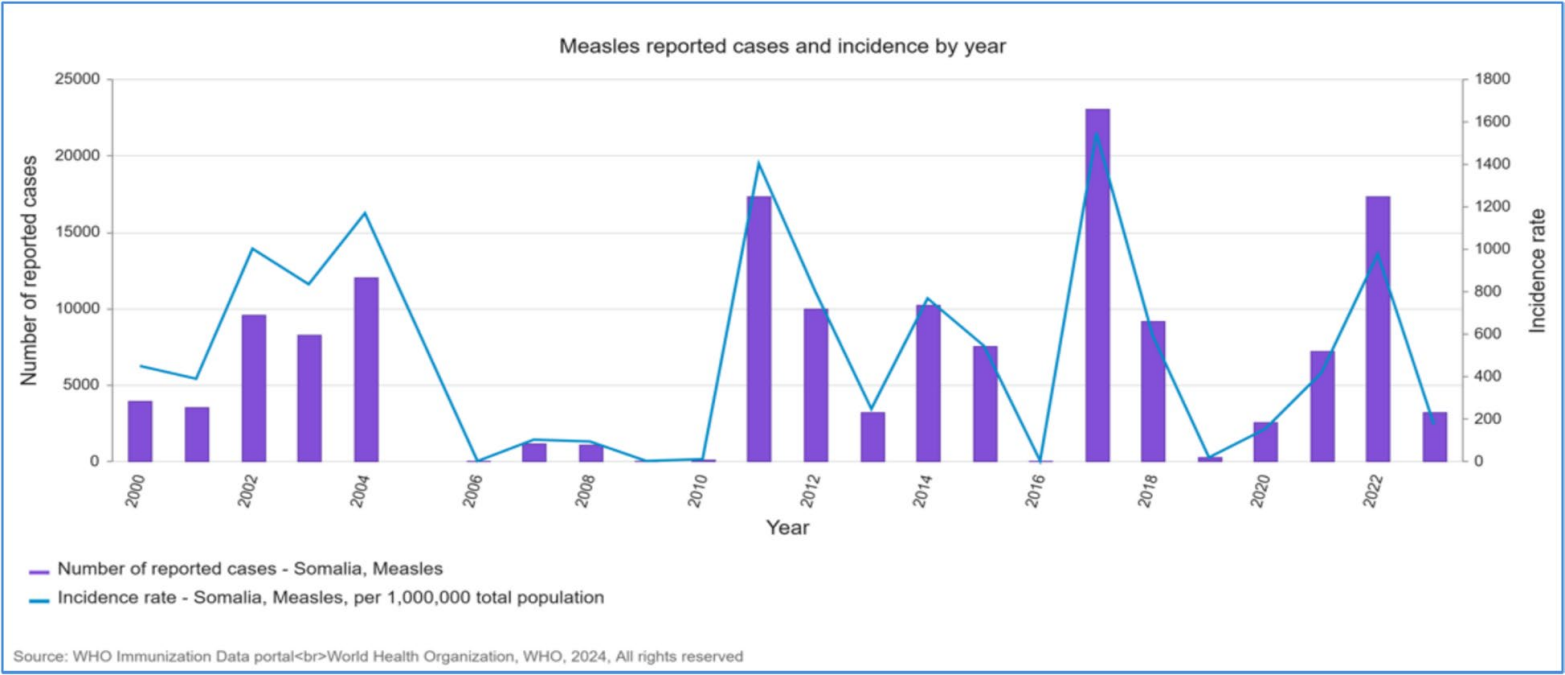
Interrelated Factors Undermining Somalia’s Health-System Resilience

Despite improvements in Integrated Disease Surveillance and Response (IDSR), challenges persist in outbreak management. The Field Epidemiology Training Program (FETP), launched in Somalia in 2021, enhances the country's outbreak detection and response capabilities. In partnership with NIH, WHO, CDC, and AFENET, FETP trains professionals in disease surveillance and management, playing a key role in the COVID-19 response and vaccine surveillance [25–27].Field Epidemiology Training Program (FETP) graduates have not fully resolved these issues [28, 29] while FETP has built considerable critical system-level weaknesses remain unresolved, specifically in expanding coverage, embedding surveillance digitally, formalizing governance, and integrating One Health approaches across Somalia [25, 28] and furthermore, reliance on international aid, covering nearly half of Somalia’s health expenditure, hampers sustainable health financing strategies [30, 31]. Figure 4 highlights the interrelated factors that undermine Somalia’s health-system resilience. This study examines the impact of climate change on infectious disease outbreaks in Somalia’s fragile health system, focusing on the interplay between environmental factors, inadequate healthcare infrastructure, and disease transmission dynamics. It aims to fill a gap in the existing literature by linking these elements, while highlighting effective control measures and case studies to strengthen response strategies. The findings will provide recommendations for improving outbreak response and developing climate-resilient public health strategies.

### Methodology: study area and study design

Somalia is a low-income, federal republic in the Horn of Africa, bordered by Ethiopia to the west, Kenya to the southwest, the Indian Ocean to the east, and Djibouti to the northwest. Somalia is divided into six federal member states (FMS) one administrative [32]. It has a population of approximately 18.1 million people [33], with a majority living in urban areas like Mogadishu, the capital [34].The research covered major cities, including Mogadishu, Berbera, Bossaso, Hargeisa, Kismayo, Merca, Baidoa, Beledweyne, Adado, Dhuusamareeb, Galkayo, and Garowe [35, 36].

The country’s vast rural areas face challenges such as poor infrastructure and limited access to healthcare. Health governance is split between a Federal Ministry of Health (FMoH) and state ministries, with service delivery dominated by primary-level health posts, maternal-and-child-health centres, Donor funds account for roughly half of total health expenditure, and human-resource density remains below the WHO minimum [30, 35]. Despite rollout of Integrated Disease Surveillance and Response (IDSR) and Field Epidemiology Training Programme (FETP) cadres, surveillance coverage is patchy, especially in rural districts and the over 2.6 million internally displaced persons (IDPs) living in over-crowded camps [28, 37]. These gaps underpin recurrent outbreaks: cholera is reported every rainy season along the Shabelle–Juba, measles remains endemic. Between 2022 and 2024, over 70,000 people died due to drought, nearly 40% of them children under five [38]. Climatic extremes drought-induced water scarcity and flood-related WASH failures further intensify fecal–oral transmission and complicate outbreak control efforts [17, 39].

**Fig. 5:**
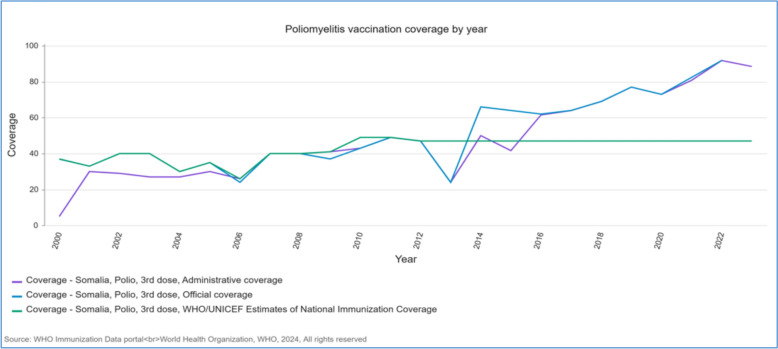
PRISMA flow diagram Selection Flowcharts

This study employed A PRISMA-guided systematic narrative review synthesized recent Somali data and case studies on cholera, measles, and polio, exposing climate-sensitive transmission patterns, revealing health-system gaps, and generating recommendations for climate-resilient outbreak response. As shown in Fig. 5, the PRISMA flow diagram illustrates the selection process for including studies in the review.

### Data collection strategy and data extraction process

This narrative review synthesizes findings from peer-reviewed articles, government reports, and grey literature on infectious disease outbreaks, health system challenges, and the impacts of climate change in Somalia. Data sources included databases such as PubMed, Scopus, Web of Science, and Google Scholar, as well as institutional websites of organizations like WHO, UNICEF, and ReliefWeb. The literature search was conducted between June 2024 and March 2025, with the final search completed on March 31, 2025. To identify studies specific to Somalia within the broader Horn of Africa context, targeted filters and keyword combinations were used, including terms such as “infectious diseases,” “climate change,” “extreme weather,” “Somalia,” and “Horn of Africa.” Literature dating from 1990 onwards was included to account for the post-civil war collapse of Somalia’s health system and to provide a comprehensive understanding of long-term Impact on infectious disease outbreaks in Somalia’s fragile health system climate-related health risks.

### Inclusion criteria

- Publications between January 1990 and March 2025.
- Studies focused on the intersection of climate change and public health in Somalia or the Horn of Africa.
- Peer-reviewed original research, government and NGO reports, systematic or narrative reviews. Studies addressing direct (e.g., infectious diseases, heat-related illnesses) or indirect (e.g., mental health, food insecurity, displacement) public health impacts.

### Exclusion criteria

- Articles published in languages other than English.
- Studies that focused solely on climate mitigation or environmental science without health-related outcomes.
- Editorials, commentaries, or opinion pieces lacking empirical evidence.

### Thematic analysis

The data were coded inductively using NVivo 12, and after applying a six-step thematic analysis (Familiarization with the Data, Generating Initial Codes, Searching for Themes, Reviewing Themes, Defining and Naming Themes and Writing the Report.

), they were synthesized into four key themes, and Two reviewers independently handled screening and data extraction, resolving differences by consensus. (1) the fragile health system, (2) control measures for infectious diseases, (3) Political Instability and Conflict, and (4) Impact of Climate Change. Each theme is explored in detail, with a focus on the challenges to have for improving public health outcomes in Somalia**.**

A study involving 202 articles was conducted through database searches (PubMed, Scopus, Web of Science, and Google Scholar) to identify relevant studies on infectious disease outbreaks, climate change, and Somalia. After removing duplicates and screening titles and abstracts, 126 articles were assessed for eligibility. After a full-text review, 74 studies were selected, providing a comprehensive view of Somalia's public health challenges, including recurring outbreaks, health system weaknesses, and the impact of climate change. The narrative review was chosen for its ability to analyze the complex relationship between climate change and infectious disease outbreaks in Somalia. The findings informed thematic analysis and recommendations for strengthening disease control and health system resilience. Future research should adopt systematic approaches to improve robustness.

## Result:

### Somalia’s health system struggles

#### Fragile health system

*Climate Impacts on Healthcare and WASH Infrastructure*: Many healthcare facilities in Somalia are poorly equipped and lack essential resources such as medical supplies, diagnostic tools, and trained personnel. This deficiency limits their ability to provide adequate care and respond to health emergencies effectively [40].Inadequate healthcare facilities and poor sanitation significantly hinder disease control in Somalia, according to the Federal Government of Somalia's Health Facility Infrastructure Assessment Report [41],many health facilities lack basic infrastructure and equipment, severely hampering effective disease control efforts. Poor sanitation is also a critical issue, as highlighted by the Somali Health and Demographic Survey, which indicates that only a small percentage of births occur in health facilities, reflecting the inadequate sanitation and healthcare infrastructure [42]. Moreover, climate change, particularly extreme weather events such as floods and droughts, exacerbates these existing gaps, further damaging infrastructure and disrupting access to clean water and sanitation. For example, flooding during heavy rains often renders healthcare facilities inaccessible and leads to the contamination of water sources, which is critical for preventing disease outbreaks [38]. The COVID-19 pandemic further strained already fragile healthcare systems by diverting staff, supplies, and surveillance resources from cholera control efforts across Africa. In Somalia, these diversions compounded long-standing WASH gaps and flood-driven exposure, highlighting how fragile health infrastructure magnifies the burden of dual outbreaks[24].

*Shortage Healthcare Personnel from Climate Change*: Somalia has an alarmingly low density of healthcare workers, with estimates indicating fewer than 0.4 doctors, nurses, and midwives per 10,000 population. This is starkly below the WHO minimum threshold of 23 per 10,000, resulting in a shortage of approximately 24,000 qualified healthcare workers needed to meet basic healthcare needs [43]. The shortage of trained health workers in Somalia limits the country’s outbreak response capabilities. The Somali Health and Demographic Survey reports that only 49% of births are attended by trained health personnel [44] due to limited infrastructure, insecurity, and cultural norms, compounded by a shortage of trained staff and financial barriers [45]. This shortage is further exacerbated by the lack of training programs and retention strategies, restricting the ability to respond effectively to health crises and outbreaks [42].The Climate change intensifies healthcare system strain through increased climate-related health issues and extreme weather events that disrupt healthcare staffing and capacity. Somalia anticipates a 1% increase in mean annual rainfall by 2030. Addressing these challenges necessitates climate-sensitive workforce strategies, such as mobile health teams and improved retention programs, to build a resilient healthcare workforce[46].

*Climate-Related Barriers to Healthcare Access*: Access to healthcare is limited, especially in displaced persons camps. Less than 30% of the Somali population has access to health services, with many facilities concentrated in urban areas, leaving rural and displaced populations underserved[47] and The Somali Health and Demographic Survey indicates that only 38% of births occur in health facilities, illustrating the widespread lack of access to essential healthcare services. Displaced persons camps face even greater challenges due to overcrowding, resource scarcity, and inadequate infrastructure, all of which are further exacerbated by climate change. Extreme weather events, such as flooding and droughts, increase displacement and disrupt already fragile healthcare systems, making it even harder for displaced communities to access essential health services [42]. The text identifies two primary impediments to healthcare access: significant out-of-pocket expenditures and the escalating impact of climate-induced migration. A substantial number of individuals are forced to fund their healthcare directly due to the limited availability of public health services, leading to financial hardship and deterring timely medical attention. This reliance on personal finances creates a cost barrier that negatively affects health-seeking behaviors. Additionally, climate-induced migration strains existing healthcare resources and imposes burdens on displaced populations. The combined pressures exacerbate existing economic and social barriers, hindering access to essential healthcare services [30].

### Control measures for infectious diseases

#### 1.2.1. Vaccination Coverage:

The insufficient vaccination coverage in Somalia poses a significant public health threat, leading to recurrent outbreaks of preventable diseases. Urgent action is needed to enhance immunization efforts, strengthen healthcare infrastructure, and increase community awareness regarding the importance of vaccinations. Studies indicate that complete immunization coverage is around 20%, which is significantly lower than the global average of nearly 80%[48], and the COVID-19 pandemic has disrupted routine immunization programs and exacerbated measles transmission in Africa, particularly in Somalia due to low vaccine coverage and a fragile healthcare system[49].

Vaccination coverage in Somalia is insufficient, leading to continued outbreaks, particularly cholera. Due in Somalia, the most frequently encountered infectious diseases are measles, cholera, and polio. Recurrent measles outbreaks, particularly affecting children under five years old, have been common since the 1990s, exacerbated by the civil war and inadequate healthcare infrastructure. Despite this, cholera, endemic in the region, resurfaces frequently due to poor access to safe drinking water and sanitation [4, 37, 50]. According to WHO and UNICEF estimates, only 49% of births are attended by trained health personnel, indicating low vaccination coverage [51].

COVID-19–driven diversions of health resources and the rise of vaccine-derived poliovirus have stalled Africa’s polio-eradication gains; in Somalia, where transmission endures in most regions and vaccination coverage is still inadequate, these external pressures heighten the risk of resurgence and reinforce the need to strengthen routine and supplementary immunization efforts [23].

#### Water and sanitation programs

The lack of access to clean water and proper sanitation contributes significantly to health issues, including preventable diseases such as malaria and severe diarrhea. High rates of infant mortality have been linked to these conditions, emphasizing the urgent need for improved WASH (Water, Sanitation, and Hygiene) services [52]. Efforts to improve water quality and sanitation face challenges due to drought and displacement. Only 52% of the population has access to a basic water supply, and 38% have access to basic sanitation facilities [42]. Prolonged drought conditions have severely reduced the availability of water resources. This has led to increased competition for limited water supplies and has exacerbated the existing deficiencies in water infrastructure [53]. Kenya’s recent outbreak confirms that inadequate WASH exacerbated by alternating floods and drought—drives persistent cholera transmission even when clinical response is swift [20]. Pan-African evidence shows that COVID-19’s resource diversion, layered onto chronically inadequate WASH infrastructure, remains the central barrier to durable cholera control[24].

### Political instability and conflict

#### Displacement

Large-scale displacement in Somalia creates overcrowded conditions, significantly increasing the risk of disease spread. There are over 2.6 million internally displaced persons (IDPs) in Somalia, many of whom live in camps with limited access to basic necessities, heightening their vulnerability to infectious diseases[54].As of early 2025 the IOM Somalia's DTM Unit, in partnership with the Danish Refugee Council (DRC), predicts that internal displacement in Somalia could reach 3.9 million by March 31, 2025, due to ongoing conflict and climate-related disasters [55], Reports indicate that 52% of newly displaced individuals are fleeing inter-clan conflict, while climate shocks remain a key driver, worsening the challenges for IDPs [56].

#### Coordination issues

The health response in Somalia is complicated by a decentralized structure where multiple organizations operate independently. This fragmentation can lead to overlapping efforts and gaps in service delivery, making it difficult to mount a unified response during outbreaks [57]. Fragmented efforts and poor coordination among stakeholders significantly hinder effective outbreak management in Somalia, leading to delays and inefficiencies in response efforts and affecting up to 30% of outbreak management initiatives [58–60].

### Impact of climate change

#### Hygiene practices

The Ministry of Environment and Climate Change (MoECC), formed in August 2022, leads the implementation of this policy, focusing on environmental protection and sustainable development [61]. Climate change exacerbates water scarcity in Somalia, severely impacting hygiene practices. With only 52% of the population having access to a basic water supply, many communities struggle to maintain essential hygiene, leading to increased rates of waterborne diseases like cholera and dysentery. According to the Ministry of Health and WHO, over the past three years, more than 900 people, most of them children under five, have died from cholera due to inadequate access to clean water [39]. Drought-induced scarcity pushes communities toward unsafe water sources, whereas floods breach sanitation systems; both conditions intensify fecal–oral transmission of Vibrio cholerae and sustain cholera outbreaks [17].

Cyclone Freddy’s floods in Malawi displaced ≈659 000 people, damaged 83 health facilities, and destroyed WASH infrastructure, driving case-fatality rates to 3% during the country’s worst recorded cholera outbreak. This climate-driven loss of safe water and sanitation parallels Somalia’s flood-prone Gu’ and Deyr seasons, underscoring how extreme rainfall events systematically amplify cholera transmission wherever WASH systems are fragile [19]. Kenya’s 2022–23 outbreak demonstrates that deficient WASH, flood contamination, and drought-induced water scarcity perpetuate cholera, supporting our claim that climate variability similarly heightens transmission risk in Somalia [20]. By tracing recent cholera surges in Ethiopia and Somalia to flood-induced WASH breakdowns, the article supplies region-specific evidence that climate variability actively drives transmission, thus buttressing our claim of climate-driven cholera risk in Somalia [24].

WASH and Healthcare Infrastructure. The effects of climate change, including frequent droughts and flooding, compromise sanitation infrastructure in Somalia. According to the Somali Health and Demographic Survey, only 38% of the population has access to basic sanitation facilities, with 28% practicing open defecation. This poor sanitation leads to the spread of infectious diseases due to improper waste disposal and environmental contamination1. In 2022 alone, 759,000 people were displaced due to drought and water scarcity, putting additional strain on limited healthcare resources and exacerbating the public health crisis [39]. The resurgence of cholera fueled by economic collapse, strained WASH services, refugee-driven overcrowding, and climate-linked water contamination mirrors the structural vulnerabilities underpinning African outbreaks, offering a useful comparative lens on how inadequate water and sanitation infrastructure perpetuates transmission [18]. While Kenya’s experience with climate-responsive WASH upgrades, decentralized surveillance, and oral cholera vaccine (OCV) drives offers a model for integrated outbreak control during floods, Somalia could benefit by tailoring similar interventions to its own climate-vulnerable districts [20]. Climate-driven displacement in IDP camps overcrowds, limiting healthcare access and facilitating disease spread [62]. Extreme weather events, like floods and droughts, damage water and sanitation systems, raising the risk of waterborne diseases such as cholera and dysentery [63, 64]. Climate variability, with fluctuating temperatures and irregular rainfall, enhances malaria vector breeding, complicating public health management [65].

### Case study selection rationale

Two case studies were selected to illustrate how context-specific, system-linked approaches have strengthened health service delivery and outbreak control in Somalia. Selection was based on three criteria: relevance to epidemic-prone settings, implementation within Somalia’s public health system, and availability of documented outcomes. Case 1 highlights adaptive vaccine delivery in insecure and displaced populations; Case 2 focuses on community-based surveillance in hard-to-reach areas.

### Case study 1: Somalia’s COVID-19 vaccination roll-out

Somalia’s inaugural campaign (March 2021) achieved only 5.5% full coverage owing to a frail health system, supply interruptions, health-worker hesitancy, fragmented federal coordination, and insecurity that limited access to internally displaced persons (IDPs) and nomads.

A revised 2022 strategy—co-designed by the Ministry of Health, WHO, and UNICEF—employed granular micro-planning, digital dashboards, 39 623 surge vaccinators, and strong community mobilization through religious and clan leaders; COVID-19 shots were co-delivered with routine childhood vaccines to maximize scarce outreach capacity.

By December 2022 the campaign had administered 8.7 million doses: 42.1% of the population (including 48% of IDPs and 16% of nomads) were fully vaccinated; women’s coverage rose from 4.0% to 46.2%; and 84 600 “zero-dose” children received their first routine immunizations. An economic analysis estimated net benefits of US $316 million against a programmed cost of US $21 million. The key challenges and strategic recommendations for strengthening Somalia’s health system are summarized in Table 1.

**Table 1:** Key Challenges and Strategic Recommendations for Strengthening Somalia’s Health System

Somalia's Health System Struggles	
Challenge	Recommendation
Fragile Health System	
1. Climate Impacts on Healthcare and WASH Infrastructure:	Invest in building and upgrading healthcare facilities, especially in rural areas
	Strengthen supply chains and partner with international organizations for support
	Establish emergency response units and stockpile critical medical resources
2. Shortage Healthcare Personnel from Climate Change:	Increase training programs and scholarships for healthcare professionals
	Improve working conditions, salaries, and incentives to retain healthcare workers
	Implement policies to incentivize rural postings and improve rural infrastructure
3. Climate-Related Barriers to Healthcare Access:	Subsidize healthcare costs and expand health insurance coverage
	Deploy mobile clinics and telemedicine services to reach underserved populations
	Train healthcare workers in cultural competency and provide multilingual services
Control measures for infectious diseases	
1. Vaccination Coverage	Launch public awareness campaigns to educate communities about vaccine benefits
	Strengthen cold chain infrastructure and partner with NGOs for distribution
	Prioritize vaccine allocation to vulnerable populations and remote areas
2. Water and Sanitation Programs	Invest in clean water infrastructure and regular water quality testing
	Conduct community education programs on hygiene and sanitation
	Develop sustainable waste disposal systems and promote recycling initiatives
Political Instability and Conflict	
1. Displacement	Provide targeted healthcare support and resources to displaced populations
	Partner with humanitarian organizations to deliver essential supplies
	Establish mental health support programs and counseling services
2. Coordination Issues	Create a centralized coordination body to streamline efforts
	Develop a unified data platform for real-time information sharing
	Simplify approval processes and secure long-term funding commitments
Impact of Climate Change	
1. Hygiene Practices	Promote community-led hygiene education programs
	Build resilient water systems and rainwater harvesting infrastructure
	Engage community leaders to advocate behavioral change
2. Poor Sanitation	Invest in climate-resilient sanitation systems
	Build and maintain public toilets and waste treatment plants
	Implement regular cleaning and maintenance of drainage systems

### The interventions include

o Tailored vaccination strategy: Community outreach and microplanning.
o Mobile vaccination teams: Reached remote areas.
o Routine vaccination integration: Combined COVID-19 with childhood vaccines.
o Surge vaccinators: Local recruits for expanded capacity.
o Mobile apps for monitoring: Real-time tracking and registration.
o Local health workers: Identified and referred individuals.
o Single-dose vaccines: Simplified vaccination process.
o Community mobilization: Engaged leaders to reduce hesitancy.

*Key lessons* tailor micro-plans to insecurity and mobility patterns; integrate real-time data tools; embed vaccination in existing primary-health and WASH platforms; and prioritise trust-building with local influencers to counter hesitancy [66].

### Case study 2: community-based surveillance (CBS) in Somalia

Since 2018 the Somaliland Red Crescent Society, with Ministry of Health & Development backing, has trained local volunteers to flag suspected cases via an SMS platform using simplified community case definitions. Coverage was extended to Awdal in 2020, targeting hard-to-reach settlements facing recurrent cholera, measles, and COVID-19 outbreaks.

In 2021 volunteers generated 138 signals, 83% of which were verified and escalated to MoHD within 24 h, triggering rapid actions (e.g., ORS distribution, health-education campaigns). Integration with routine ICCM activities broadened service reach and reinforced community trust.

### The interventions include

o CBS by SRCS: Detects and responds to disease outbreaks in communities.
o Nyss platform: Collects real-time data and sends alerts via SMS.
o CHV training: Volunteers trained to recognize and report diseases.
o MoHD collaboration: Verifies and responds to health alerts.
o Integration with health programs: Enhances disease detection and response.

*Key Lessons* Local ownership; Low-tech reporting; Speed matters; Adaptability; System linkage Capability; System linkage[67].

## Discussion

This review examines how climate variability, conflict-related fragility, and systemic service gaps sustain recurrent cholera, measles, and polio outbreaks in Somalia. Using a PRISMA-guided narrative synthesis of 72 sources, four case studies, and two Somali program evaluations, we identified four key themes: (1) fragile health system, (2) control measures for infectious diseases, (3) political instability and conflict, and (4) impact of climate change. We also identified interconnected weaknesses, including critical infrastructure and workforce deficits, suboptimal vaccination coverage, inadequate WASH services exacerbated by climate shocks, fragmented outbreak coordination, and external disruptions, particularly COVID-19, which diverts resources from routine control.

Somalia’s health-facility density and human-resource ratios remain among the lowest globally, with < 0.4 physicians + nurses + midwives per 10 000 population—twenty-three-fol Complete EPI coverage hovers near 20%, leaving a persistent pool of susceptible for measles and cVDPV2 [48]. The pandemic further disrupted supply chains, diverted cold-chain capacity, and fuelled vaccine hesitancy among health workers—processes documented continent-wide [49] and linked to stalled polio eradication [23]. Somalia’s 2021–22 COVID-19 campaign nonetheless demonstrated that context-specific micro-planning, surge vaccinators, and integration with routine outreach can raise full-course coverage above 40% in one year—even in insecure districts [66]. Embedding these tactics into routine EPI (e.g., quarterly “integrated outreach weeks” that bundle measles, polio, and OCV with nutrition and antenatal care) could close the immunity gap while reducing marginal delivery costs.

The compounded effects of Somalia’s fragile health infrastructure and climate variability—marked by recurrent droughts, floods, and displacement—directly undermine disease control by degrading WASH systems, overwhelming health facilities, and intensifying the transmission of waterborne and vector-borne diseases. These findings affirm that climate change is not a peripheral concern but a central determinant of health in Somalia, driving increased cholera outbreaks through flood-induced contamination and exacerbating healthcare inaccessibility in drought-stricken and displaced populations [17, 18, 63].

Only 52% of Somalis access basic water and 38% basic sanitation [39, 42]. Drought concentrates communities around unsafe sources, while cyclical Gu’ and Deyr floods breach latrines and spread Vibrio cholerae, creating an uninterrupted faeco-oral pathway [17]. Regional precedents corroborate this mechanism: Lebanon’s 2022 cholera resurgence despite emergency OCV [17], Malawi’s cyclone-driven WASH collapse with a 3% CFR [18], and Kenya’s 2022–23 flood-linked spikes controlled only after rapid WASH repair and county-level OCV drives [20]. These experiences show that vaccines alone are insufficient when climate shocks repeatedly compromise water systems. Somalia therefore needs drought-proof boreholes, raised latrine platforms, and flood-resistant pipe networks co-financed through the national climate-adaptation plan and humanitarian pooled funds. Real-time hydro-meteorological dashboards, linked to IDSR alerts, should trigger pre-emptive chlorination and OCV in high-risk riverine districts [69].

The health response in Somalia is further complicated by a decentralized system, where multiple organizations often operate independently. This fragmentation results in overlapping efforts and gaps in service delivery, which hinders the efficiency of health interventions. The federal architecture in Somalia distributes outbreak authority across six member states and numerous partners, creating a scenario of duplicated assessments and delays in the release of essential commodities, as noted in previous studies [57–59, 70]. In contrast, Kenya’s incident-command model, which features joint RDT stockpiles, weekly epidemiological situation reports (sit-reps), and WASH-plus-vaccine task forces, has proven effective in reducing cholera case fatality rates (CFRs) below 1% [20]. This model provides a useful framework for Somalia to adopt through the establishment of a National Outbreak Operations Centre (NOOC). This center could serve as a centralized platform to aggregate state-level data and facilitate timely surge deployments, addressing the slow response times and improving coordination across all regions. By strengthening the integration of resources and coordinating health initiatives, Somalia could enhance its preparedness and response to public health threats, especially in the context of climate-induced disease outbreaks [71]. A promising solution to Somalia’s fragmented outbreak response lies in scalable, community-level innovations. For example, Somaliland’s SMS-based Community-Based Surveillance (CBS) system successfully verified 83% of 138 alerts within 24 h and integrated smoothly with ICCM services [67]. Scaling this low-tech, volunteer-driven model nationwide could address core challenges—such as delayed outbreak detection, limited lab capacity, and poor WASH oversight—by accelerating early warnings and improving community accountability [72, 73]. When linked with a centralized National Outbreak Operations Centre, as inspired by Kenya’s incident-command model, CBS could play a pivotal role in aggregating grassroots data, triggering faster response deployments, and harmonizing federal and state-level surveillance efforts.

A narrative design allowed the integration of peer-reviewed studies, grey reports, and operational field notes sources essential in fragile contexts with sparse indexed literature. Future systematic reviews and meta-analyses, combined with primary implementation research on community-owned interventions, are needed to refine burden estimates and intervention cost-effectiveness.

## Conclusion

Somalia’s recurrent cholera, measles and polio outbreaks stem not only from fragile infrastructure and political instability but are now decisively amplified by climate change, whose alternating droughts and flash floods degrade water-sanitation systems, concentrate populations around unsafe wells, contaminate riverine sources, and swell overcrowded IDP camps where low immunization coverage lets pathogens flourish. To break this climate–outbreak feedback loop, disease-control efforts must explicitly internalize climate risk by protecting and climate-proofing WASH facilities (drought-resilient boreholes, flood-raised latrines, pre-positioned chlorination supplies), embedding vaccination and surveillance in mobile, weather-adaptive outreach models, establishing a National Outbreak Operations Centre that fuses meteorological, environmental and epidemiological data for anticipatory action, and ring-fencing domestic and donor financing for climate-resilient health programmes rather than relying on post-crisis appeals.

Future research should quantify the fraction of outbreak burden attributable to specific climate hazards and evaluate the cost-effectiveness of anticipatory versus reactive interventions, supported by dashboards that overlay disease incidence, coverage gaps and seasonal forecasts. Without this dual agenda of health-system strengthening and climate adaptation, each extreme-weather event will erase hard-won gains; conversely, a climate-resilient, community-centred health system offers Somalia its most reliable path to protecting vulnerable populations from the infectious-disease threats of a warming world.

## Pathway recommendations

To address the ongoing challenges of infectious disease outbreaks in Somalia, policymakers should prioritize the following actions and we propose the following sequential implementation pathway:

### Phase 1 – strengthen healthcare infrastructure

#### Lead actors: federal MoH, regional health bureaus, WHO, international donors

Mobilize financing Convene federal, regional and donor partners to secure dedicated budgets and grants.

Upgrade and equip facilities Renovate primary health posts in rural and IDP sites; supply essential drugs, cold‐chain equipment and emergency kits.

Bolster workforce capacity Recruit, train and deploy multidisciplinary teams (clinicians, lab technicians, community health workers) with emphasis on hard-to-reach areas.

### Phase 2 – expand community-based immunization

#### Lead actors: MoH, UNICEF, red crescent, local NGOs, clan and religious leaders

Micro-planning and stakeholder engagement Map underserved communities; partner with local leaders, NGOs and women’s groups to drive ownership.

Deploy integrated outreach teams Launch mobile vaccination “days” combining routine EPI, supplemental campaigns (measles, polio) and catch-up immunizations.

Monitor and adapt Establish real-time reporting dashboards; conduct quarterly coverage reviews to identify gaps and reallocate resources.

### Phase 3 – roll out climate-resilient WASH programs

#### Lead actors: ministry of environment and climate change (MoECC), MoH, UN-Habitat, WASH-focused NGOs

Conduct climate-vulnerability assessments Identify flood- and drought-prone zones; characterize water-borne disease hotspots.

Construct and rehabilitate WASH infrastructure Build flood-proof water points, latrines and hand-washing stations; retrofit existing systems for drought resilience.

Integrate early-warning and community training.

## Data Availability

Not applicable.

## Abbreviations

IDSR: Integrated disease surveillance and response
FETP: Field epidemiology training program
WHO: World Health Organization
UNICEF: United Nations Children’s Fund
WASH: Water, sanitation, and hygiene
PRISMA: Preferred reporting items for systematic reviews and meta-analyses
NGO: Non-governmental organization
WASH: Water, sanitation, and hygiene
IDP: Internally displaced person
MOH: Ministry of health
HIV: Human immunodeficiency virus
AIDS: Acquired immunodeficiency syndrome
MCH: Maternal and child health
TB: Tuberculosis
IPD: Inpatient department
HRH: Human resources for health
EPI: Expanded programme on immunization
